# Electro-Therapeutics

**Published:** 1896-12

**Authors:** Charles H. Rosenthal

**Affiliations:** Cincinnati, Ohio


### Electro-Therapeutics.

BY CHARLES H. ROSENTHAL, D. D. S., CINCINNATI, OHIO.

Read before the Knapp Dental Coterie, Ft. Wayne, Ind., December 3, 1896.

Appreciating the great value of a pain-obtundant for dental operations, I placed in my office about a year ago an electro-galvanic apparatus, especially well-adapted for making cataphoric applications. To give you the results of my efforts in this line is the object of this paper. If I were to give you only the results of my first fifteen or twenty cases, so encouraging were they that I could have enthusiastically recommended cataphoresis, and would have even gone so far as to say that no dentist should practice without an apparatus. To my regret, however, I soon learned that these first cases happened to be the first victims of my anxiety to try this—then new—device. The cases were poorly selected. If the electrodes without current or remedial agents had been employed, and I had simply told the patient of the wonders that were to be performed, as much good would have been done. Subsequent experiment bears this out with reference to those cases where such an application would prove of real value; in those cases of sensitive dentine where patient and operator alike could be benefited, I put myself on record as not having had a single perfectly satisfactory result. The remedial agents employed were McK. & R. Guai Cocaine, as recommended by the *Cosmos*. Also the pure mur. of cocaine, each with the same unsatisfactory result in cases of sensitive dentine.

The only practical use as a local anæsthetic which is at all satisfactory, is its application to expose pulps where, in the judgment of the operator, extirpation of the pulp is indicated. This may be done by placing a small crystal of pure cocaine in immediate contact with the pulp, and applying from one-half to one and a half milliamperes of current; the current should be allowed to play from fifteen to twenty minutes. This will insure a painless removal, obviating the use of the time-honored arsenious acid with its dangers and inconveniences, the pulp-canal being left in an aseptic condition and ready, at once, to be filled.

Its use for stopping toothache, in cases of exposed pulp, is to be discouraged if extirpation of the pulp is not intended, as it would only be a question of a short time, were the pulp capped after cataphoric treatment of such an exposed pulp, that it would die—due to the destructive effect of the galvanic current on the tissues themselves.

Of the antiseptic action of the galvanic current, considered in relation to the treatment of pulpless teeth, there is much to commend it. The positive pole is a good germicide, decomposing, as it does, all organic matter with which it comes in contact—used in connection with the saturated solution of sodium chloride, the reaction setting free nascent chlorine applied to the tooth, will permeate the most tortuous root-canal, thoroughly sterilizing the most septic conditions. Three to five milliamperes, applied with a fine platina electrode, from fifteen to twenty minutes, suffices. It is advisable to immediately fill the root-canal before removing the rubber dam, avoiding the possibility of rendering the parts septic by contact with the ever-present germs of the mouth.

Having given this method a thorough test, extending over several months, and not having met with a single unfavorable result, I take pleasure in submitting this to your consideration.

My mode of filling root-canals, after this treatment, is to whittle a piece of orange-wood to a very fine point, notching it so that it will break off about the length of the root; soak in the tincture of iodine until the pores are thoroughly saturated; mix, of a pasty consistency, iodoform and glycerine, placing the same on the end of the stick, carrying to the root-canal and applying with a churning motion. Break it off, and allow to remain in the tooth, covering the end with cement.

